# Environmental impact and cost of bio-based hydrophobic multifunctional coatings

**DOI:** 10.1007/s11356-026-37701-3

**Published:** 2026-04-07

**Authors:** Pooja Yadav, Paula Nousiainen, Muhammad Farooq

**Affiliations:** 1 Natural Resources Institute Finland, Bioeconomy and Environment / Sustainability Assessments Unit: Luonnonvarakeskus (Luke), Latokartanonkaari 9, 00790 Helsinki, Finland; 2 Aalto University, School of Chemical Engineering, Department of Bioproducts and Biosystems, Vuorimiehentie 1, 02150 Espoo, Finland

**Keywords:** Betulin, Lignin, Suberin, Environmental impact and cost, Hydrophobic coating, Life cycle assessment

## Abstract

The circular bioeconomy supports climate action by promoting the use of renewable materials in sustainable production. In this study, bio-based betulin, lignin, and suberin were used as raw materials for producing the multifunctional hydrophobic coatings. Life cycle assessment (LCA) was used to study the environmental impact of these protective coatings from cradle to gate. The foreground data were collected from laboratory experiments and literature, while background data were sourced from the ecoinvent 3.10 database. The functional unit (FU) used was coating production and application on 1 m^2^ of fabric. The environmental impacts and cost were evaluated using Recipe (H) 2016 midpoint method in SimaPro 9.6. The results indicated that per FU, the global warming potential (GWP) was 2.92 kg CO_2_ eq. for suberin coating, 2.39 kg CO_2_ eq. for betulin coating, and 2.01 kg CO_2_ eq. for lignin coating. The sensitivity analysis was conducted and indicated that replacing ethanol with bioethanol reduced the burden on GWP and fossil resource scarcity (FRS) but increased the burden on land use (LU), terrestrial ecotoxicity (TE), and human non-carcinogenic toxicity (HNCT). Additionally, the source of energy in the process, particularly the participation of nuclear and bio-based electricity, was found to influence the results on GWP, IR, and LU impact categories. The recycling rate of solvents and the production process of feedstocks (suberin, betulin, and lignin) significantly impacted the results.

**Supplementary Information:**

The online version contains supplementary material available at 10.1007/s11356-026-37701-3.

## Introduction

Advancing bio-based chemical production methods to replace fossil-derived alternatives is vital for addressing the contemporary global challenges (Harman-Ware et al. 2021). Forest biomass is a sustainable and renewable resource, but it has limitations to use, and for that reason, more efficient utilization of biomass is required (Oettel and Lapin 2021; Yadav et al. 2024). Factors such as available land, materials, and other sustainability constraints limit the amount of biomass that can be responsibly extracted (Carlqvist et al. 2022). Bark is a lignocellulosic material, and it has attracted interest in recent years for its potential in various value-added utilization (Kwan et al. 2022). The extractives found in barks offer potential uses in various applications, including surface coatings, textiles, and food ingredients (Almeida et al. 2019; Kwan et al. 2022). Bark of birch is an important byproduct of biorefineries and has mainly been used for energy production (Yadav et al. 2024). It also holds great potential as a source of bioactive compounds, including betulin, lignin, suberin, oleanolic acid, and lupeol (Zhao et al. 2020). Suberin and betulin are potential materials for multifunctional hydrophobic coatings for textiles sectors and packaging (Kumar et al. 2022). Lignin is inherently amphiphilic, rather than strongly hydrophobic, and requires modification for effective use in hydrophobic applications (Ruwoldt et al. 2023).

In birch outer bark, suberin constitutes the largest proportion of bark components, reaching up to 45% by weight of its solid matter (Kumar et al. 2022; Yadav et al. 2024). While suberin cannot be extracted in its intact form, it can be broken down via alkaline hydrolysis, methanolysis, and ionic liquid extraction (Li et al. 2016). Suberin is a hydrophobic biopolymer; its potential industrial application has been explored in previously published studies (Quilter et al. 2024). Suberin has the potential to be used in hydrophobic coatings production (Kumar et al. 2022). In addition to suberin, the outer bark of silver birch contains 24.5% betulin by weight (Demets 2022; Yadav et al. 2024), which is co-extracted with suberin during processing. Betulin, the most abundant triterpenoid of the lupan series, possesses valuable properties such as pharmacological, antiviral, antitumor, antibacterial, hypolipidemic, and hydrophobic character (Demets et al. 2022; Yadav et al. 2024). Betulin-derived compounds offer eco-friendly alternatives for producing water-repellent textiles (Huang et al. 2019).

Lignin is a natural resource, and the second most abundant plant-based biopolymer material on Earth, as well as the most abundant aromatic biopolymer (Priyadarshi et al. 2024). Lignin is generated as a by-product of the pulp and paper industry, as well as biorefineries, with an estimated annual production of 50 million tons (Mobredi et al. 2024; Priyadarshi et al. 2024). Currently, around 1–2% of the annual lignin produced is used for the development of value-added products, while the remainder serves as a fuel source for energy generation in power plants (Mobredi et al. 2024). Due to its amphiphilic nature, lignin holds potential for use in multifunctional coatings applications (Souza et al. 2019). Several research studies have explored the wettability of coatings based on lignin. Numerous studies have investigated the wettability of lignin-based coatings, revealing that different types of lignin display varying degrees of hydrophobicity (Mobredi et al. 2024). Additionally, lignin’s inherent UV-protection, antimicrobial resistance, and antioxidant activity provide beneficial multifunctional properties to the coating applications (Ruwoldt et al. 2023).

The textile industry increasingly seeks sustainable alternatives to conventional hydrophobic coatings, which are often derived from petroleum-based chemicals. Bio-based hydrophobic coatings for fabrics offer an eco-friendly solution by utilizing renewable resources. These coatings provide water repellence while maintaining breathability and comfort, which are crucial for applications in clothing, upholstery, and technical textiles (Babaeipour et al. 2024a, b; Khan et al. 2022). Combining permeability and waterproofness in a garment creates a material with two main functions that somewhat contradict each other (Babaeipour et al. 2024a, b). The development and implementation of renewable bio-based fluorine-free formulations for hydrophobic coating treatments can reduce the adverse environmental and biological impacts typically associated with the synthesis of conventional liquid-repellent coatings (Mates et al. 2016).

Over the past few decades, various methods have been developed to fabricate hydrophobic surfaces, but synthesis processes of these coatings frequently involve toxic organic solvents (Tang et al. 2013; Mates et al. 2016), complicated processing methods, and use of fluorinated chemistries (Gao & He 2013). However, the previously published methods are not practically feasible for large-scale commercial applications (Mates et al. 2016), and the environmental impact of these processes is not available. Duan et al. (2016) studied chitosan-based coatings, a biopolymer from chitin, known for its potential in fabric coatings. Chitosan can be chemically modified to improve its hydrophobic properties. Sehaqui et al. (2014) investigated the use of cellulose nanocrystals from plant cellulose to create hydrophobic coatings for fabric substrates, enhancing water repellence. Rahmadhani et al. (2024) developed a nanocomposite coating using chitosan and silicon dioxide (SiO₂) to impart hydrophobic and antibacterial properties to textiles. The SiO₂ was derived from rice husk ash, while the chitosan was extracted from crustacean shells.

It is important to assess the environmental impact of hydrophobic coating treatments and their production process before advancing to large-scale research or commercial applications. Climate change is a global challenge of increasing concern that has prompted action across multiple sectors, including the European Union (EU). However, addressing the sustainability of new technologies, such as the production of bio-based hydrophobic coatings, is essential to ensure they contribute positively to sustainable development goals. It requires a thorough quantitative environmental impact analysis, and the Life Cycle Assessment (LCA) is a tool that is increasingly being used to assess and compare environmental impacts of products and processes at their early stage (Fidan et al. 2024). Yadav et al. (2024) evaluated the environmental load of producing betulin and suberin from birch bark using extraction and alkaline hydrolysis methods. Lecart et al. (2023) evaluated the impact of suberin coating. Bernier et al. (2013), Hermansson et al. (2020), and Kumaniaev et al. (2020) have conducted LCA and examined the environmental impacts of kraft lignin production as a by-product or main product from biorefineries that are used for fuels.

As per our knowledge, none of the previously published studies used suberin, botulin, and lignin bio-based materials to produce the multifunctional hydrophobic coatings and assess their environmental impacts and costs. This study is the first to assess the environmental sustainability of three different bio-based coatings derived from suberin, betulin, and lignin. These coatings were compared based on environmental impact and cost to identify the environmentally friendly coatings overall from material selection to self-assembly or production of nanoparticles (NPs) and then their application process. These coatings have the potential to replace fossil-based coatings, such as Teflon, used for textiles. The contribution analysis was conducted to identify the environmental hotspots within the process. This study will help to find out the way to reduce the environmental burden of production and application of coatings by performing the sensitivity analysis using different energy sources and bio-based solvents. The reliability of the data and results was confirmed by conducting an uncertainty analysis using the Monte Carlo simulation features in the SimaPro 9.6 software, utilizing primary and secondary data.

## Materials and methods

### Goal and scope definition

This study investigates the environmental implications across the life cycle of betulin, lignin, and suberin-based hydrophobic coatings for textile and packaging applications.

#### Functional unit

The functional unit (FU) is a measurable and clearly defined reference for normalization and is determined in the context of LCA. Plehn et al. (2012) discussed the FU in the manufacturing system, a traditional approach applied to evaluate design alternatives of simple products. For outdoor wall paint, FUs are used as a reference for the function of the investigated item covering 1 m^2^ of surface with paint or coating (Plehn et al. 2012). Therefore, in this study, 1 m^2^ FU is used, allowing for the possibility of comparison with the literature. Overall, 1 m^2^ of bio-based coating on cotton fabric materials was used as FU, which required 2.86 L of coating solution, as per our laboratory experiments.

#### System boundary

System boundaries start from raw materials that were obtained from the forest, with the production of suberin and betulin from the outer bark of birch hardwood and kraft lignin as a residue from a softwood kraft pulp mill. The process involves the production of betulin, suberin, and lignin, the production of NPs and is followed by the application of coating dispersion on fabric. The “cradle-to-gate” system boundary was followed for hydrophobic functional coating material production, with a focus on its application phase.

### Life cycle inventory analysis

The primary data collection for the life cycle inventory (LCI) primarily relied on laboratory experiments conducted at Aalto University (Table S1). Additional data were obtained from scientific publications and the ecoinvent 3.10 database, which provided secondary data for the Finnish electricity mix, ethanol production, acetone, water, and other chemical inputs (Table S2).

#### Kraft lignin production

Lignin was received from UPM (Lappeenranta, Finland) as part of the support and collaboration for this project. The lignin was produced using the kraft pulping process from softwood and purified according to the company’s general protocol. The obtained kraft lignin was dried in powder form and was of the grade UPM BioPiva™ 395. The water content of the product was 5%. The material was used as such without further purification or drying for preparation of lignin nanoparticles (*LNPs*). The kraft process is the most dominant process in pulp and paper industries and is considered a traditional method to separate lignin from the lignocellulosic biomass (Bernier et al. 2013; Gordobil et al. 2021; Bilal et al. 2021). Figure S1 shows the lignin production process from kraft pulp and input data presented in Table S2. Mass allocation was used to calculate the environmental impact of kraft lignin in this study, following the procedure described in detail by Bernier et al. (2013).

#### Betulin and suberin production

Suberin hydrolysate and betulin fraction were obtained from Natural Resources Institute Finland (LUKE). Both fractions were isolated from hardwood birch (*Betula pendula*) stems. The procedure is described in detail in the study by Yadav et al. (2024) and Fig. S2. In short, silver birch (Punkaharju, Finland) outer bark was milled using a Fritsch Pulverisette cutting mill (Fritsch GmbH, Germany). The powder was subjected to ethanol extraction and subsequent alkaline ethanolic hydrolysis. The extraction in ethanol: water was done in a 2.0-L Büchi Glas Uster stirred autoclave (Büchi AG, Uster, Switzerland) at 90 °C, and isolated by evaporation under reduced pressure. The residual extracted bark was hydrolyzed according to Korpinen et al. (2019). Ethanol and 20 w% NaOH in water was used in the reactor at 90 °C for 60 min, and the hydrolysate was collected and evaporated. The residue was washed with boiling water to isolate additional betulin fraction to yield a combined betulinol-rich residue (BF) approximately 36% of the charged fractionated outer bark. The filtrate was finally acidified to pH 4 to precipitate out water insoluble suberin fatty acids. The yield of the suberin fatty acid rich fraction (SH) was 26% calculated from original charged outer bark. The details of conducting LCA for production of the suberin and betulin were described in the study by Yadav et al. (2024).

##### Allocation

**Fig. 1:**
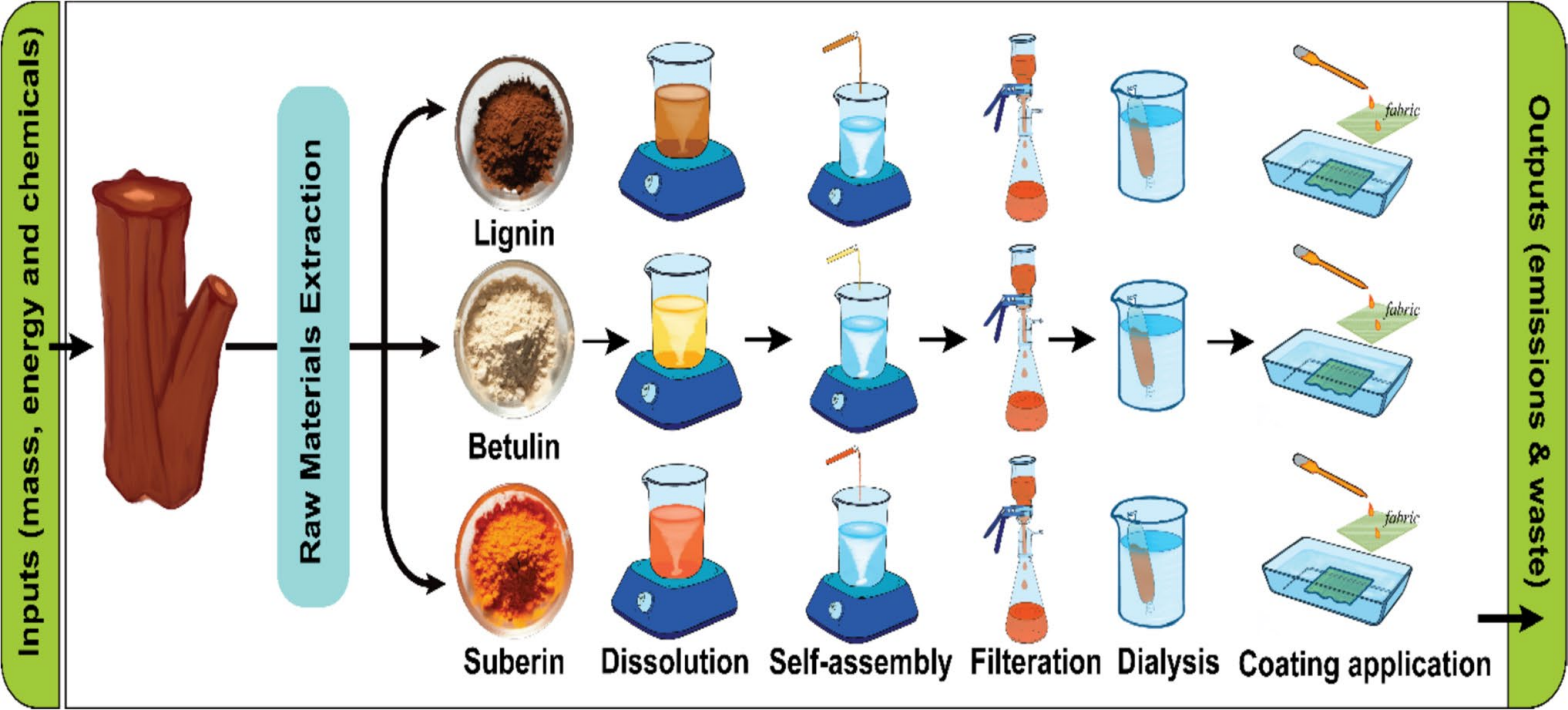
Schematic representation of the production process (system boundary) for suberin, betulin, and lignin nanoparticle dispersion coatings and their application onto cotton fabric

According to ISO 14044, allocation should be avoided. However, in this study, allocation cannot be avoided, and allocation was based on mass shares that reflect the physical relationships between the products and co-products throughout the product system. In this study, system expansion was applied by subtracting the avoided impacts from using bark residue for energy. Birch bark was assumed to represent 13.80% of log volume, with 25% outer bark and equal basic densities for bark and wood (500 kg/m^3^), so allocations were based on mass shares. As shown in Fig. 1, betulin I and extraction bark residue were allocated by mass (16.5% and 83.5%, respectively). Allocation between betulin II and suberin after precipitation was based on solid content (3.90% and 96.10%). The credits from using hydrolyzed bark residue for district heating were subtracted, and energy credits were assigned according to the calorific value of woody materials (Piccinno et al. 2016). Finnish mix electricity was used as the source of energy in the process. It was assumed that a 98% ethanol recycling rate (Yadav et al. 2021a, b), with an energy requirement of 0.586 kWh/kg ethanol (Pfeffer et al. 2007). Comprehensive information on the production of suberin and betulin is provided in the study “Life Cycle Assessment of Suberin and BetulinProduction from Birch Bark” by Yadav et al. (2024).

#### Nanoparticle formation (self-assembly) from betulin, lignin, and suberin

Self-assembly of lignin to produce *LNPs* was performed according to Table 1. following the solvent exchange method by Zou et al. (2019). Shortly, 1 w% of kraft lignin was dissolved in acetone for 1 h. The solution was then filtered using a paper filter (Whatman, pore size 0.7 μm) to remove any undissolved residues. Spherical particles were formed through self-assembly by pouring the solution into vigorously stirred deionized water at a 1:3 (v/v) ratio. Acetone was removed 99% from the LNP using rotary evaporation at 40 °C under reduced pressure. Supramolecular self-assembly of suberin-rich hydrolysate (SH) and betulin-rich fraction (BF*)* solutions containing 1 wt% of SH and BF in acetone were formulated according to Table 1. The mixtures were initially agitated at 600 rpm for 15 min at 65 °C, ensued by constant stirring at ambient temperature for 1 h to facilitate dissolution. The solutions were centrifuged at 10,000 rpm for 30 min to eliminate any undissolved residues. Self-assembly was achieved by rapidly transferring the dilutions or mixtures into deionized water that was vortex-stirred, with a solution-to-water ratio of 1:5 (v/v). Subsequently, the dispersed particles underwent dialysis against water for approximately 48 h using Spectra/Por 1 tubing with a molecular weight cutoff (MWCO) of 6–8 kDa to eliminate the organic solvent. Dilutions and mixtures in this study were prepared using freshly made solutions, following the established protocol to prevent crystallization. Although dialysis is commonly used in nanoparticle preparation, it was not applied here. Instead, acetone was removed via rotary evaporation, which is more scalable and avoids the high-water consumption and long processing times associated with dialysis, by improving the feasibility of industrial implementation.

**Table 1:** Inputs and outputs of nanoparticle formation, coating material formation, and application of coating solution on fabric

Materials	Lignin solution	Betulin solution	Suberin solution	Unit	Source/reference
Lignin (dry matter)	0.1	-	-	g	UPM BioPiva 395
Betulin (dry matter)	-	0.1	-	g	Laboratory (Luke)
Suberin (dry matter)	-	-	0.1	g	Laboratory (Luke)
Acetone (concentration, 100%)	10	10	10	ml	VWR
Stirring (10 min at 65 °C)	-	0.65	0.65	kcal	1000-W stirrer
Stirring for 1 h	1	1	1	kWh	1000-W stirrer for 1 h
Total	10	10	10	ml	
Nanoparticle formation (self-assembly)					
Deionized water	7	7	7	ml	
Lignin Solution	2	-	-	ml	
Betulin solution	-	2	-	ml	
Suberin solution	-	-	2	ml	
Acetone recycling rate	99	99	99	%	Ashok et al. (2018) and Rivière et al. (2021)
Electricity for recycling of acetone	0.11	0.11	0.11	kWh/kg	Capello et al. (2005)
Filtration (cellulose membrane)	12.5	12.5	12.5	cm	Marchery-Nagel
Cellulose membrane	1.05	1.05	1.05	gm	Weigh of membrane
Acetone removal bio-membrane	2	2	2	cm	need membrane
Total nanoparticle dispersion	7	7	7	ml	
Coating application process for 5-cm (***L***) and 3.5-cm (***W***) fabric					
Ethanol (concentration 96%)	1	1	1	ml	Anora Group, Finland
Deionized water	3	3	3	ml	
Cationic starch solution (concentration, 0.1 wt.%)	5	5	5	ml	Chemigate Oy, Finland
Coating dispersion solution	5	5	5	ml	
Total	14	14	14	ml	

#### Application of coating (based on suberin, betulin, and lignin) on fabric

Cotton fabric was cut into small strips (5 × 3.5 cm), then cleaned with ethanol and deionized water to remove any possible contaminants. The strips were soaked in water for a few minutes prior to the layer-by-layer deposition process. Each strip was immersed in a 0.1 wt% cationic starch solution for 5 min, followed by three rinses with deionized water to ensure uniform deposition of the cationic polyelectrolyte and to eliminate loosely bound molecules. The strips were then immersed in NPs dispersions for 20 min, followed by rinsing to remove unabsorbed particles. This dip-coating process was repeated layer by layer until two bilayers were formed on the cotton fabric. Finally, the coated samples were dried at room temperature. The TENCEL™ fabric samples measuring 5 cm × 3.5 cm were excised and washed with ethanol, followed by a rinse with deionized water to eliminate any potential contaminants present on the fabric. Subsequently, fabric samples were immersed in a solution of 0.1 wt% cationic starch for 20 min. Following this, the fabric was rinsed for a further 5 min with deionized water. The fabric was immersed in NPs dispersions for 20 min, followed by a subsequent rinsing for 5 min. To ensure adequate coverage, the fabric specimens were left to dry overnight. Subsequently, a second layer of the dispersion was applied, followed by a 5-min rinse with deionized water.

### Life cycle impact assessment

The life-cycle environmental impact was calculated using ReCiPe 2016 Midpoint (H) V1.09/World (2010) H method (Huijbregts et al. 2017) and is well suited for global prospective (Huijbregts et al. 2017). The environmental impacts were calculated on global warming potential (GWP), ionizing radiation (IR), ozone formation (OF), fine particulate matter formation (FPMF), freshwater eutrophication (FWE), terrestrial acidification (TA), freshwater ecotoxicity (FE), terrestrial ecotoxicity (TE), human carcinogenic toxicity (HCT), marine ecotoxicity (ME), human non-carcinogenic toxicity (HNC), mineral resource scarcity (MRS), fossil resource scarcity (FRS), land use (LU), and water consumption (WC). The background data were adapted from the database Ecoinvent 3.10 using the cutoff model (Table 1). It is important to discuss biogenic carbon when dealing with wood-based biomass products, and for that reason, the Environmental Footprint (E.F) 3.1 (Andreasi et al. 2023) method was used for calculating the biogenic carbon emissions. The energy demand in the process was calculated using the Cumulative Energy Demand (LHV) V1.01 method (Frischknecht et al. 2007).

### Environmental and production cost

The environmental and production cost analysis was performed to identify the most economical scenario. The capital investment costs were associated with high uncertainty; for that reason, the analysis primarily focused on environmental cost and production cost. The production costs for suberin, betulin, and lignin-based coatings were estimated based on the inputs (chemicals, mass, and energy balance calculations) (Thunman et al. 2019; Yadav et al. 2020, 2021a). The raw material prices were as follows: suberin at 25 €/kg (CORDIS 2024), betulin at 570 €/kg (NST Chemicals 2024), and kraft lignin at 380 €/t (Bajwa et al. 2019). The prices of acetone (1253 €/t) and ethanol (805 €/t) were obtained from Kuittinen et al. (2022), while water was priced at 2.52 €/m^3^ and bioethanol price was 776 €/t (Yadav et al. 2021a, b; Chembid 2020). The electricity price of 0.089 €/kWh was sourced from Eurostat (2023). Production costs were estimated for 1 m^2^ of coating material produced from suberin, betulin, and lignin consistent with the FU. The production cost excluded VAT and taxes, labor cost, and subsidies, and added in details in supplementary.

The environmental price for each environmental indicator was assessed at the midpoint level, as reported by CE Delft (2024). The environmental prices were determined by multiplying the environmental impact of each impact category by its external costs (Shanmugam et al. 2019; Yadav et al. 2021b). In this study, external costs for all impact categories were considered at midpoint level from the Environmental Prices Handbook 2024: EU27 (CE Delft 2024) in detail mentioned in Table S3. External costs study the possible damage to society when 1 kg of additional pollutant finds its way into the environment (CE Delft 2024).

### Sensitivity analysis

The sensitivity assessment was performed by replacing the (a) source of electricity, (b) replacing the bioethanol instead of fossil ethanol, and (c) modeling ethanol recovery in the process by assuming a 98% ethanol recycling rate (Yadav et al. 2021a, b; Yadav et al. 2024) and an energy requirement of 0.586 kWh per kilogram of ethanol recovered (Pfeffer et al. 2007). This assumption is important because ethanol recovery efficiency is associated with energy use that strongly influences the overall environmental impact of suberin, betulin, and lignin coating production and application.

### Uncertainty analysis

The manufacturing of coatings affects different unit processes, causing uncertainty in data. To evaluate uncertainties of all three coating productions and applications, uncertainty analysis was done using Monte Carlo simulations in SimaPro 9.6 using a 95% confidence interval (SimaPro 2025; Yadav et al. 2021b) using the life cycle inventory data (water, electricity, acetone, and ethanol) for the production of a hydrophobic coating solution of 2.86 L that was applied on FU. A semi-quantitative approach was employed using the Ecoinvent 3.10 database, where ratings were assigned based on five data quality indicators: completeness, reliability, temporal relevance, geographical correlation, and further technological applicability (Yadav et al. 2021b; Patel and Singh 2024). For assessing the uncertainty of all inputs (electricity, water, acetone, and ethanol) in the process, a pedigree number between 1 and 5 was used to indicate the quality of data in SimaPro 9.6 using the Ecoinvent database 3.10. In the database, a parameter score of 1 symbolizes excellent or reliable data quality, although a score of 5 indicates poor data quality (Ciroth et al. 2012). For example, in the case of electricity, the assigned scores were (1, 1, 1, 1, 1, and not applicable). This indicates that the data quality used in this study was reliable. A score of 1 means the data is verified and based on direct measurements. Completeness: The score was also 1, meaning the data is representative of all relevant sites for the market considered. Temporal correlation: A score of 1 indicates that the data is less than 3 years different from the time of the dataset. Geographical correlation: A score of 1 signifies that the data originates from the area under study. Technological correlation: A score of 1 reflects that the data comes from companies, materials, and processes under study. The sample size was unspecified, which is why it was marked as “not applicable.” To derive measures of uncertainty, 1000 iterations were performed using ReCiPe (*H*) midpoint environmental impact categories.

## Results and discussion

The selection of sustainable solutions, both environmental and economic, is crucial in developing a new bio-based product for the market. As part of this, the environmental impact of suberin, betulin, and lignin coatings was evaluated across seventeen environmental indicators. The environmental impact on GWP of suberin coating was 2.92 kg CO_2_ eq., for betulin coating 2.39 kg CO_2_ eq., and for lignin coating 2.01 kg CO_2_ eq. per FU. The environmental impacts on other impact categories per FU are presented in Table 2. The lab-scale coating application process requires chemicals such as cationic starch solution, ethanol, and water. For this reason, the environmental impact of producing 1 m^2^ of coating has also been calculated and it is presented in Table 2. The GWP of suberin coating without application per 1 m^2^ was 1.99 kg CO_2_ eq., which was 31.85% lower than per FU. Betulin coating without application per 1 m^2^ was 1.46 kg CO_2_ eq. that was 38.91% lower than per FU. The GWP of lignin coating without application per m^2^ was 1.08 kg CO_2_ eq., that was 46.26% lower than per FU.

**Table 2:** Environmental impact of different coating material production with and without the application of coating on fabric at lab scale

Impact categories	Unit	Suberin coating production	Betulin coating production	Lignin coating production	Suberin coating per FU	Betulin coating per FU	Lignin coating per FU
GWP	kg CO_2_ eq	1.985	1.455	1.078	2.916	2.386	2.009
IR	kBq Co-60 eq	2.248	1.619	0.907	2.274	1.646	0.934
OF	kg NO_x_ eq	0.003	0.003	0.002	0.005	0.005	0.004
FPMF	kg PM^2.5^ eq	0.003	0.002	0.001	0.004	0.003	0.002
TA	kg SO_2_ eq	0.008	0.004	0.003	0.011	0.006	0.005
FWE	kg P eq	0.001	0.000	0.000	0.001	0.001	0.001
TE	kg 1.4 DCB	9.420	8.693	8.685	11.555	10.828	10.820
FE	kg 1.4 DCB	0.028	0.022	0.017	0.035	0.029	0.024
ME	kg 1.4 DCB	0.046	0.037	0.030	0.055	0.046	0.039
HCT	kg 1.4 DCB	0.058	0.046	0.035	0.074	0.062	0.051
HNCT	kg 1.4 DCB	1.303	0.989	0.645	1.548	1.235	0.891
LU	m^2^∙a crop eq	0.330	0.171	0.117	0.804	0.645	0.591
MRS	kg Cu eq	0.003	0.002	0.002	0.003	0.003	0.002
FRS	kg oil eq	0.600	0.439	0.331	1.112	0.950	0.842
WC	m^3^	0.245	0.214	0.239	0.277	0.247	0.239
GW biogenic	kg CO_2_ eq	0.003	0.004	0.003	0.003	0.004	0.003
CED (LHV)	MJ	84.07	59.53	39.39	111.93	86.81	66.68

*GWP* global warming potential, *IR* ionizing radiation, *OF* ozone formation, *FPMF* fine particulate matter formation, *TA* terrestrial acidification, *FWE* freshwater eutrophication, *TE* terrestrial ecotoxicity, *FE* freshwater ecotoxicity, *ME* marine ecotoxicity, *HCT* human carcinogenic toxicity, *HNC* human non-carcinogenic toxicity, *LU* land use, *MRS* mineral resource scarcity, *FRS* fossil resource scarcity, *WC* water consumption, *GW* global warming biogenic, *CED* cumulative energy demand (LHV)

The production of suberin and betulin-based coatings was found to have higher environmental impacts than lignin-based coatings. The betulin coating had 27% lower GWP than the suberin coating. The betulin coating production had lower environmental impacts on IR (28%), OF (11%), FPMF (40%), TA (54%), FWE (29%), TE (8%), FE (21%), ME (19%), HCT (21%), HNCT (24%), LU (48%), MRS (21%), FRS (27%), and WC (12%) impact categories compared to the suberin-based coating. The environmental impacts of the lignin-based coating on GWP (46%), IR (60%), OF (39%), FPMF (60%), TA (67%), FWE (57%), TE (8%), FE (40%), ME (35%), HCT (40%), HNCT (51%), LU (65%), MRS (42%), FRS (45%), and WC (2%) were lower than the suberin coating. While the betulin coating showed lower water consumption than the lignin coating (Table 2). However, the biogenic carbon dioxide emissions of all three coatings are approximately similar (Table 2). The environmental impacts of these coatings are highly dependent on their production methods and production of raw materials, key assumptions, including functional equivalence, allocation methodology, and laboratory-scale processing. Specifically, the environmental impact observed in the present study is influenced by the production process and maturity of technology associated with suberin, betulin, and kraft lignin. However, there are still several issues that need to be overcome before the industrialized production of suberin and betulin can be realized, as detailed in our previous LCA study (Yadav et al. 2024), since the environmental impact of betulin and suberin production is extended from the study by Yadav et al. (2024). However, the production of kraft lignin is an established technology with relatively low environmental impact (Bernier et al. 2013). The results of this study are based on the environmental impact, while the quality of hydrophobicity and functionality of the coating plays an important role in the decision-making factor, which has not been covered in this study.

When assessing the environmental effect of any process, it is essential to evaluate its energy efficiency. The energy required for the process is calculated using the cumulative energy demand (CED) method based on the lower heating value (LHV). The CED consists of a combination of non-renewable (77.31%) and renewable (22.69%) energy sources to produce all three coatings. The non-renewable component includes fossil fuels, nuclear energy, and biomass, while the renewable component comprises biomass, wind, solar, water, and geothermal energy. Suberin coating production required 84.07 MJ energy, betulin (59.53 MJ), and lignin required 39.9 MJ.

**Fig. 2:**
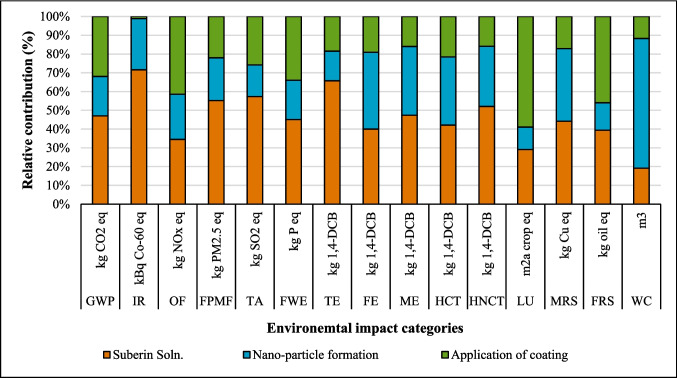
Environmental impact of suberin coating per functional unit based on the contribution of different stages

**Fig. 3:**
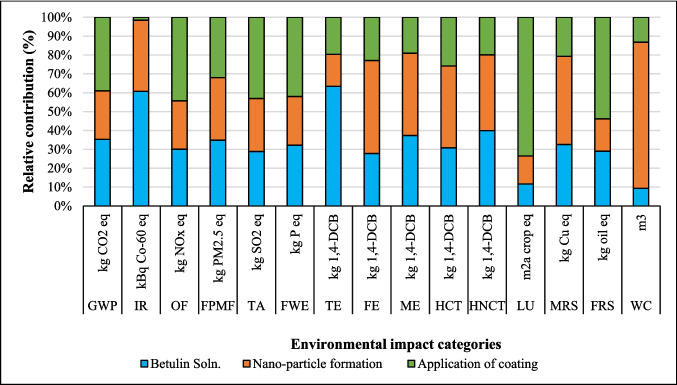
Environmental impact of betulin coating per functional unit based on the contribution of different stages

**Fig. 4:**
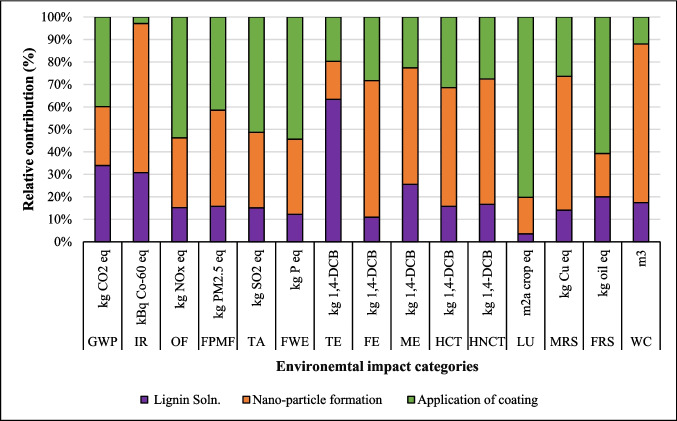
Environmental impact of lignin coating per functional unit based on the contribution of different stages

The relative contribution of environmental impact categories across different stages of coatings production, including dissolution of suberin, betulin, and lignin, NPs, and application of coating onto fabric, is presented in Figs. 2, 3, and 4. The findings indicated that the environmental impacts of the NPs and coating application stages are approximately the same for all three coatings due to identical inputs and outputs in these processes. However, the suberin dissolution stage GWP is 1.37 kg CO_2_ eq. that is higher than the GWP of *LNPs* formation and application of coating on fabric, and the same trend is observed for IR, TE, FPMF, TE, HCT, and HNCT impact categories. The contribution analysis shows that the GWP of betulin (0.84 kg CO_2_ eq.) and lignin (0.47 kg CO_2_ eq.) solutions are higher than that of NPs formation but lower than the coating application stage (Figs. 3 and 4 and Table S4). When comparing the dissolution stages of the three coatings, the betulin dissolution stage had a higher environmental impact than lignin but lower than suberin. This was due to the production processes of suberin, betulin, and lignin. Especially, the suberin and betulin production processes were intensive in terms of ethanol, energy, and water usage (Fig. S2). The environmental indicators to produce betulin and suberin depend on the process’s mass and energy balance, the energy source, the ethanol recycling rate, and the type of allocation method employed. An efficient industrial process could reduce the GWP of suberin (Fig. S2) and betulin (Yadav et al. 2024).

In the lignin coating production process, lignin dissolution had a GWP of 0.47 kg CO_2_ eq., which was lower than the environmental impacts of NPs formation (0.61 kg CO_2_ eq.) and application stages (0.93 kg CO_2_ eq.). This was attributed to the lignin solution formation phase that required 0.65 kcal less energy compared to suberin and betulin, which required 0.65 kcal per 10 ml solution for stirring for 10 min at 85 °C (Table 1). This lower energy requirement is one reason for the reduced environmental impact of lignin coatings compared to betulin and suberin coatings. The lignin used in this study is a byproduct of the pulp industry, as shown in Fig. S1, with inventory data sourced from the study by Bernier et al. (2013) presented in Table S1. The use of lignin as a renewable feedstock in coating production successfully lowers the environmental impact compared to suberin and betulin on the considered environmental impact categories. The choice of renewable feedstocks (lignin, betulin, and suberin) significantly influences the environmental outcomes of the process and emerges as a key determinant affecting the results. The environmental impacts of suberin, betulin, and lignin are contingent upon their production methods, as influenced by the system boundaries, allocation strategies employed, efficiency of the process, size of scale, and quality of data used. The contribution analysis outcomes for lignin-based coatings are illustrated in Fig. 4.

The results of other materials in the market that are used for hydrophobic coating in the textile industry are compared with our study results. The previously published studies were found to focus mainly on the GWP. The commonly used hydrophobic material in the market, Teflon, has a GWP of 13.11 kg CO_2_ eq. per m^2^ (Gore and Associates 2013) application that is higher than the GWP values of this study’s coatings [suberin (2.92 kg CO_2_/FU), betulin (1.38 kg CO_2_/FU), and lignin (2.01 kg CO_2_/FU)]. Silicones have a GWP of 1.88 kg CO_2_/m^2^ (Althaus et al. 2007), which is lower than suberin (1.99 kg CO_2_/m^2^) but higher than betulin (1.46 kg CO_2_/m^2^) and lignin (1.07 kg CO_2_/m^2^). While silicon-based and Teflon coatings production is well established, our results based on lab-scale experiments for suberin, betulin, and lignin-based coatings may evolve as the technology matures. For that reason, it is important that the observed GWP advantage of the bio‑based coatings must be interpreted with caution. The comparison between the bio‑based coatings and the commercial Teflon and silicone coatings considers only GWP due to data limitations, and the system boundaries are not scale‑consistent. Specifically, when the commercial coatings represent mature industrial processes, whereas the bio‑based coatings are modeled at laboratory scale, which can lead to inflated impacts per unit of product as well as potential overestimation of relative environmental benefits. A full, multi‑impact comparison using harmonized, industrial‑scale system boundaries would be necessary to draw definitive conclusions about their comparative environmental performance.

### Results of contribution analysis

**Fig. 5:**
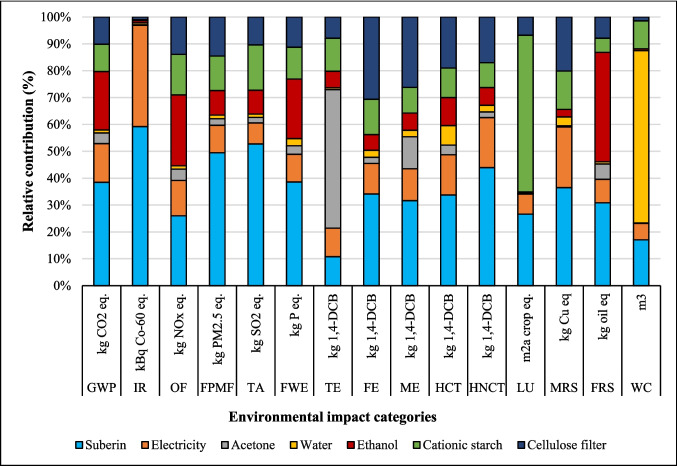
Results of contribution analysis of different inputs used in the process to production of suberin coating per FU

Environmental hotspots were identified by analyzing the contributions of components such as suberin, betulin, lignin, electricity, acetone, ethanol, cationic starch, water, and filters in the process. The results of the contribution assessment of different components used per FU for suberin coating are presented in Fig. 5. The production of suberin raw material (1.12 kg CO_2_ eq.) was the main contributor to GWP, followed by the use of ethanol (0.63 kg CO_2_ eq.) during the application stage. Electricity use was the third highest contributor (0.42 kg CO_2_ eq.), and water had the lowest impact (0.03 kg CO_2_ eq.) among all components used in the coating production and application process. In addition, cationic starch (0.30 kg CO_2_ eq.), filter paper (0.29 kg CO_2_ eq.), and acetone (0.12 kg CO_2_ eq.) had moderate contributions to GWP.

Suberin was the main contributor to IR (1.35 kBq Co-60 eq.), FPMF (0.002 kg PM^2.5^ eq.), TA (0.006 kg SO_2_ eq.), FEW (0.004 kg P eq.), MRS (0.0012 kg Cu eq.), HCT (0.025 kg 1,4-DCB), HNCT (0.68 kg 1,4-DCB), and LU (0.21 m^2^∙a crop eq.) impact categories (Table S5). The use of chemicals like ethanol and sulfuric acid in suberin production significantly contributes to environmental impacts in various categories. Electricity was the main contributor to IR and MRS impact categories, primarily due to the use of Finnish mix electricity. Acetone, on the other hand, contributed to the TE impact category due to its production process.

**Fig. 6:**
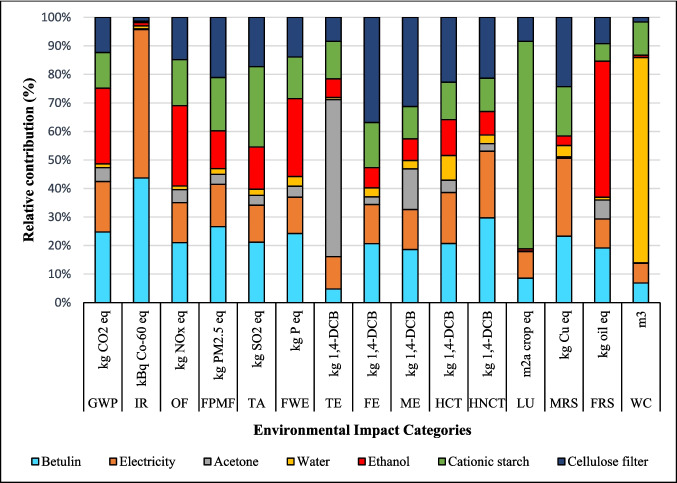
Results of contribution analysis of different inputs used in the process to production of betulin coating per FU

The environmental impacts varied significantly across different input materials and processes. Ethanol emerged as the primary contributor to GWP, OF, and FRS, while betulin was the second highest contributor to GWP, IR, OF, FPMF, FE, and FRS (Fig. 6). Electricity consumption predominantly influenced IR and MRS, while showing minimal impact on other categories. LU was primarily driven by cationic starch production, followed by betulin, cellulosic filter, and electricity consumption (Table S6). These LU impacts can be attributed to the cultivation of raw materials required for starch, cellulose, and betulin production, as well as the Finnish electricity mix. WC was directly impacted by process water requirements.

**Fig. 7:**
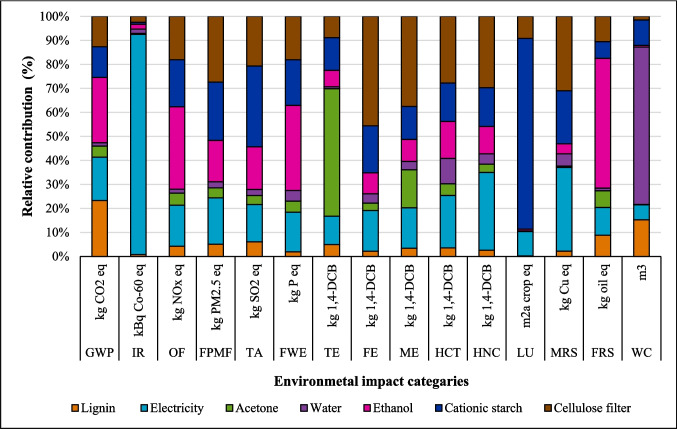
Results of contribution analysis of different inputs used in the process to produce lignin coating per FU

The environmental impact contributions of different components in the lignin-based coating and application process are shown in Fig. 7 and Table S7. Ethanol was the primary contributor to GWP, while acetone dominated TE impacts. Water consumption directly influenced WC, and cationic starch was the main driver of LU. Lignin production significantly affected the IR, HCT, and HNCT categories.

### Results of sensitivity analysis

#### Change in source of electricity

**Fig. 8:**
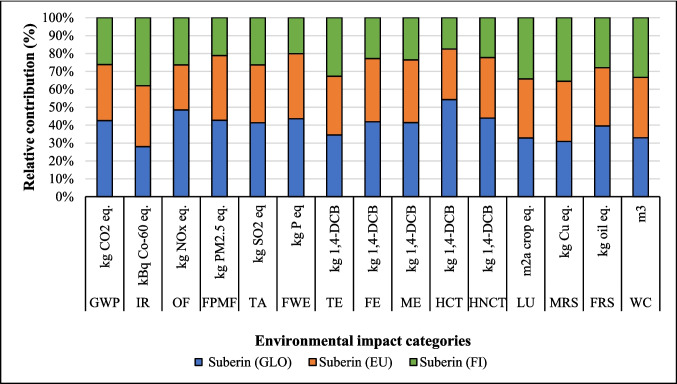
Changes in the results suberin coating per FU by changing Finnish average mix (FI) electricity to European average mix (EU) or global mix (GLO)

**Fig. 9:**
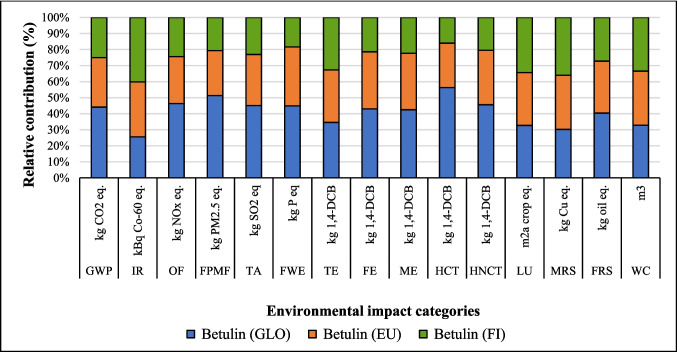
Changes in the results of betulin coating per FU by changing Finnish average mix electricity (FI) to European average mix (EU) and global mix (GLO)

**Fig. 10:**
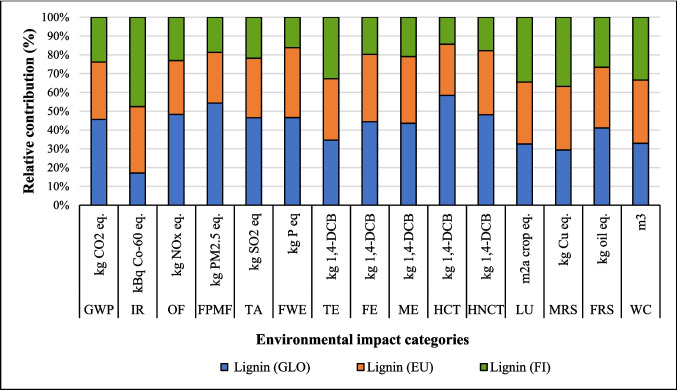
Changes in the results production of lignin coating by changing Finnish average mix (FI) electricity to European average mix (EU) or global mix (GLO)

The LCA outcomes are primarily influenced by two key factors: the source of electricity and the type of ethanol used in the process. Figures 8, 9, and 10 show that the highest GWP occurred when the global (GLO) electricity mix (Table S8) was used, whereas the lowest GWP was observed with the Finnish (FI) electricity mix. The reason is the high percentage of electricity generation from coal at a global level. In contrast, the Finnish electricity mix has a lower percentage of fossil and coal-based electricity and a higher percentage of renewable electricity (Energy statistics Finland 2023). Finland’s electricity mix comprises biomass (25%), nuclear power (27.4%), hydropower (19.2%), wind power (9.7%), solar power (0.3%), and imported electricity (18.4%) from Russia and the remainder from Sweden (Energy statistics Finland 2023; Yadav et al. 2024). Sweden generates 48% of its electricity from nuclear power, while 64% of Russia’s electricity is produced from fossil fuels (EMBER 2023). Among all the coatings, the highest impact on the IRP category was observed with the use of nuclear power in Finland’s electricity grid mix. In contrast, the application of GLO electricity resulted in the lowest impact within the IRP category (Fig. 8).

The percentage changes in environmental impact when Finnish mix electricity was replaced by Europe (EU) and global (GLO) electricity mixes are shown in Table S8. In suberin coating, by replacing FI by EU and GLO mix electricity, GWP increased by 63% and 19%, respectively. Conversely, IR and LU impact categories were reduced by 26% and 11%, respectively, with the GLO and EU mixes. The same trends were observed for betulin and lignin coatings in GWP, IR and LU impact categories (Table S8). Europe’s power grid composition is 40% renewable energy, 38.6% fossil fuels, and 20% nuclear power and others. Natural gas is the main fossil energy source used to generate electricity (19.6%), followed by coal (15.8%) (Europa 2023). Globally, fossil fuels remain the highest source of electricity generation, with the GLO mix grid composition being different from the EU power grid. The coal-based energy is 35.5%, while natural gas follows with a 23% share, and China, India, and the United States accounted for the largest contributors (STATISTA 2023). For lignin coating, GWP increased by 28% and 91% when FI was replaced by EU and GLO electricity, respectively. HCTP increased by 91%, whereas IRP decreased 64% by replacing FI by GLO electricity, respectively (Table S9).

#### Bioethanol as an alternative to ethanol

**Fig. 11:**
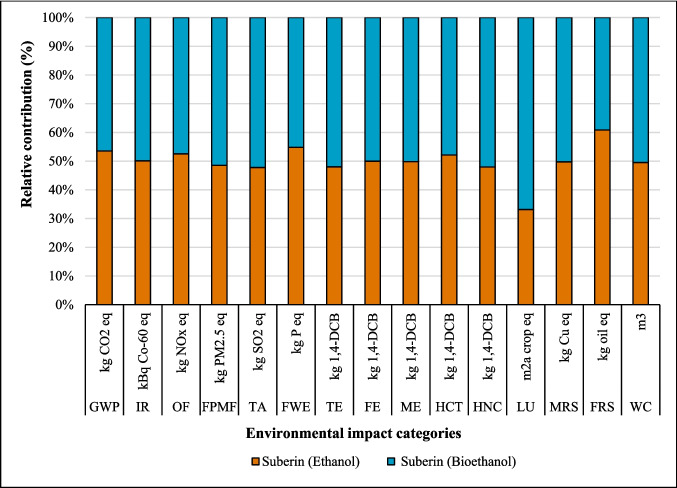
Changes in the results production of suberin coating by changing ethanol to bioethanol per FU

**Fig. 12:**
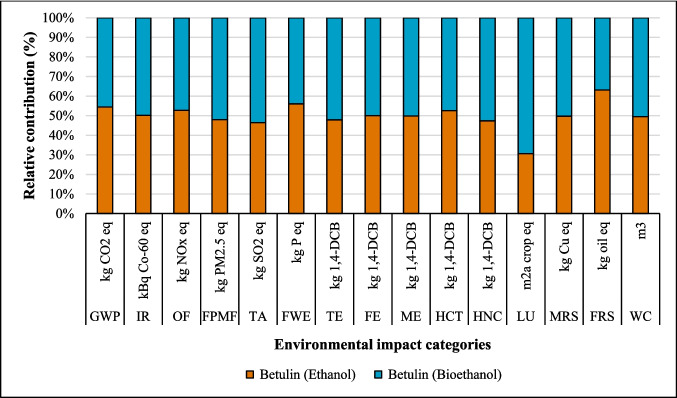
Changes in the results production of betulin coating by changing ethanol to bioethanol per FU

**Fig. 13:**
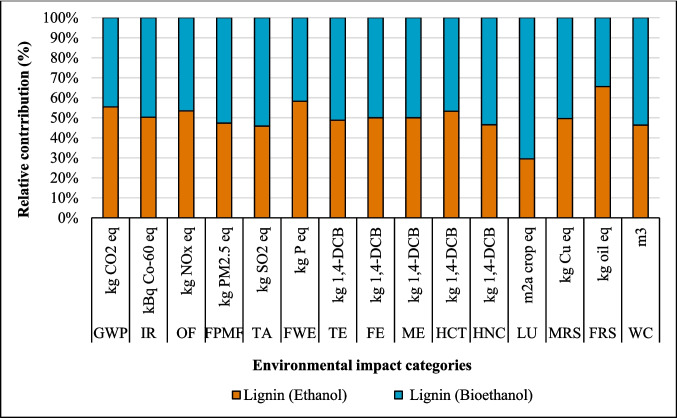
Changes in the results production of lignin coating by changing ethanol to bioethanol per FU

The substitution of fossil ethanol with bioethanol demonstrated varying environmental impacts across the three coating systems. A modest reduction in GWP was observed for suberin coating, decreasing from 2.92 to 2.53 kg CO_2_, with similar trends noted for betulin and lignin coatings (Figs. 11, 12, and 13). While minimal changes were observed in IR, FE, and ME impact categories, significant variations emerged in LU, FRS, TA, and WC. Notably, LUP increased substantially by 102% (from 0.80 to 1.62 m^2^∙a crop) due to wood and agricultural land requirements in bioethanol production. This increase in LU impacts was consistent across both the betulin and lignin coating systems. The trade-off indicates that although the use of bioethanol lowers GWP, this benefit is offset by substantially higher LU driven by competition for agricultural land, pressures on soil quality, and constraints in biomass availability. Therefore, the GWP advantage of bioethanol must be interpreted in the context of these land-related challenges, including concerns about biomass supply and competition with food, feed, and forestry production. Detailed comparative results for all impact categories are presented in Table S10.

#### Effect of ethanol recovery (ER)

**Fig. 14:**
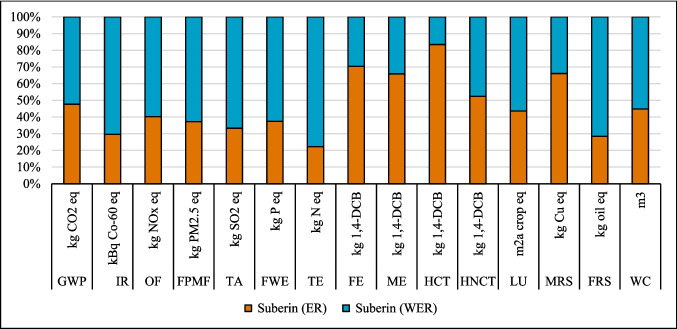
Changes in the results production of suberin coating by changing ethanol recovery per FU. Ethanol recovery (ER), without ethanol recovery (WER)

**Fig. 15:**
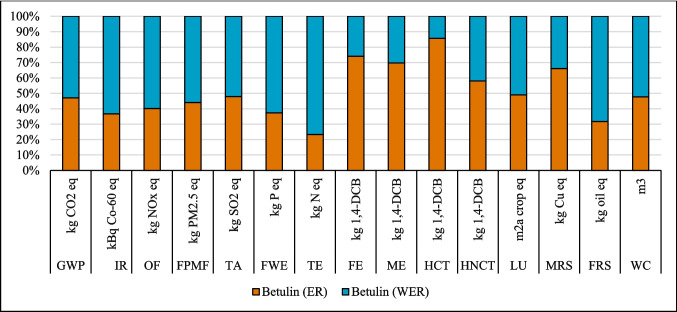
Changes in the results production of betulin coating by changing ethanol recovery per FU. Ethanol recovery (ER), without ethanol recovery (WER)

**Fig. 16:**
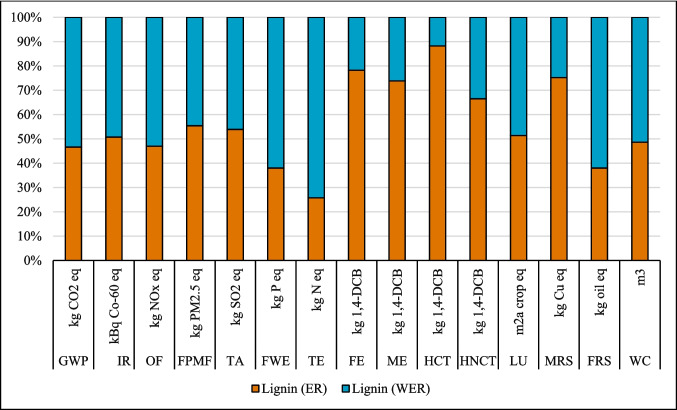
Changes in the results production of lignin coating by changing ethanol recovery per FU. Ethanol recovery (ER), without ethanol recovery (WER)

The analysis demonstrated that variations in ethanol recovery during the production and application phases of the suberin, betulin, and lignin coatings significantly influenced the environmental impact results, as shown in Figs. 14, 15, and 16. High ethanol recycling rate substantially reduces the demand for virgin ethanol, while the energy required for solvent recovery directly contributes to the system’s energy-related burdens. Consequently, even small changes in recycling efficiency or energy demand can noticeably alter environmental impacts across different categories. An ethanol recycling rate of 98% in the process reduced impacts in the following categories for the suberin coating: GWP (26%), IR (132%), OF (0.2%), FPMF (0.2%), TA (1%), TE (825%), LU (18%), and FRS (67%), with similar trends observed for the betulin and lignin coatings (Figs. 15 and 16). These reductions are attributed to lower refinery emissions, decreased fossil energy use, reduced solvent losses, and an overall decrease in resource consumption. In contrast, impacts on FE, ME, HCT, and MRS increased due to the additional energy required for ethanol recycling.

### Production cost and environmental cost

Since the betulin, suberin, and lignin coating production processes are precommercial; for that reason, industrial data is unavailable and capital and investment costs are highly uncertain at the trial stage. Both production and environmental costs vary according to the raw materials utilized in each coating. Environmental costs were calculated by multiplying the impact of each environmental indicator by its associated externality cost (results listed in Table S11).

The external cost for suberin coating is 1.50 €/kg per FU, for betulin coating is 1.20 €/kg per FU and for lignin coating is 0.96 €/kg per FU, while without coating application is 1.10 €/kg for suberin, 0.80 €/kg for betulin, and 0.57 €/kg for lignin. Environmental cost is highly dependent on external prices of FPMF, which is 99.2 €/kg PM^2.5^ eq., whether the environmental impact on FPMF was low for all coatings and did not significantly influence the external costs of coatings.

The production cost of suberin is 2.21 €/kg, for betulin is 1.23 €/kg, and for lignin is 0.20 €/kg (Table S12). Suberin and betulin have higher market prices in comparison to lignin because the production of suberin and betulin raw material is more costly than lignin. This is the main reason for the higher prices of suberin and betulin coatings. The production of lignin is a well-established industrial process, which lowers its price significantly. Table S12 also shows the changes in price per FU, with and without the application stage. Ethanol is only needed at the application stage and for that reason the cost is provided in two parts: one is production cost without application, and another is total cost per FU. The price is also influenced by other components such as electricity, bark, and other chemicals used in the process.

### Uncertainty analysis

The results of the uncertainty analysis provide insight into the reliability and quality of the data across various parameters, as outlined in the methodology section (Uncertainty analysis Sect.). Detailed results of the uncertainty analysis were reported in Table S13 on different impact categories, including the mean, median, standard deviation (SD), coefficient of variation (CV), and standard error of the mean (SEM). In general, CV less than 10% is considered a good stability indicator, whereas more than 10% indicates that the results are more variable. The results showed that CV values for GWP were within ± 10%, while the values for LU were higher than 10%. A high SD indicates that the data is widely spread, making it less reliable, whereas a low SD suggests that the data is closely clustered around the mean, indicating greater reliability, as shown in Table S13. The SD values range from 0.15 to 10.18 across various impact categories. The GWP category had an SD of 0.15, indicating high precision and consistency in the results. In contrast, the WC and HNCT impact categories showed SD values greater than 10, suggesting higher variability and less reliability.

### Limitation of the study

This assessment is limited to a cradle-to-gate system boundary and does not include use-phase durability or end-of-life behavior. Therefore, the present results cannot claim substitution of fossil-based coatings without confirmation of comparable lifetime performance. In our previous work on LNP-based coatings, we demonstrated key functionalities such as UV shielding, antibacterial properties, water repellency, stain resistance, and maintained durability under washing and rubbing even after two washing cycles implying robust attachment of the particles to the fabric surfaces (Babaeipour et al. 2024a, b). Recently, the recyclability (end-of-life) of the LNPs and LNPs mixed with wax particle coatings has also been assessed together with techno-economic analysis and market assessment (Guillen et al. 2026). Moreover, the suberin and betulin-based particle coatings multifunctional properties have also been evaluated showing excellent hydrophobicity, anti-staining, and antimicrobial activity (Farooq et al. 2025). However, this study remains limited to a cradle-to-gate, and a full cradle-to-grave life-cycle assessment, complemented by extended durability protocols (multiple laundering and abrasion cycles, UV/weathering) and end-of-life scenario analysis, would be highly beneficial to significantly advance this area and to provide a clearer picture of coating performance in practical and commercial contexts.

## Conclusions

This LCA indicates that wood‑derived hydrophobic coatings based on suberin, betulin, and lignin show strong environmental performance and may be suitable sustainable substitutes for fossil‑based coatings like Teflon, although having some limitations. It was found energy source selection emerged as a critical factor, with the Finnish electricity mix offering environmental advantages due to its low fossil fuel dependency. While bioethanol substitution showed promise in reducing global warming potential. Further optimization through renewable energy integration presents opportunities for enhancing the environmental performance of these bio-based coating systems. The production cost and environmental cost of the three coatings were calculated and found production cost depends on the inputs used in the process and their market price while environmental cost depends on the emissions produced during the coating’s production and application. The environmental impact assessment, encompassing both production and economic dimensions, revealed complex interrelationships between process variables and environmental outcomes. It was found that the study’s results are highly dependent on key assumptions, including functional equivalence, allocation methodology, and laboratory‑scale processing, which collectively limit the generalizability of the findings.

## Supplementary Information

Below is the link to the electronic supplementary material.

ESM 1 (DOCX 728 KB)

## Data Availability

Data will be made available upon reasonable request.
