# Supplementary material for: Environmental impact and cost of bio-based hydrophobic multifunctional coatings
Source: Environ Sci Pollut Res Int. 2026 Apr 7;33(14):6657–77. doi: 10.1007/s11356-026-37701-3 (PMC13124888; doi:10.1007/s11356-026-37701-3)
Supplement: Supplementary file 1 — (DOCX 728 KB) [file 11356_2026_37701_MOESM1_ESM.docx]

**Supplementary Material**

**Environmental Impact and Cost of Bio-based Hydrophobic Multifunctional Coatings**

**by**

Pooja Yadav^1*^, Paula Nousiainen^2^, Muhammad Farooq^2^

^1^Natural Resources Institute Finland (Luke), 00790 Helsinki, Finland

(email: pooja.yadav@luke.fi) *Corresponding author.

^2^Aalto University, Vuorimiehentie 1, 02150 Espoo, Finland.


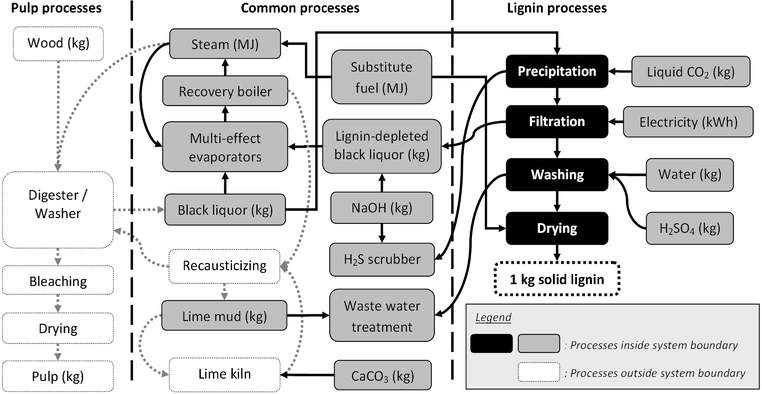


**Figure S1**: System boundary for the kraft lignin life cycle inventory considered in present paper (Bernier et al. 2013).

**
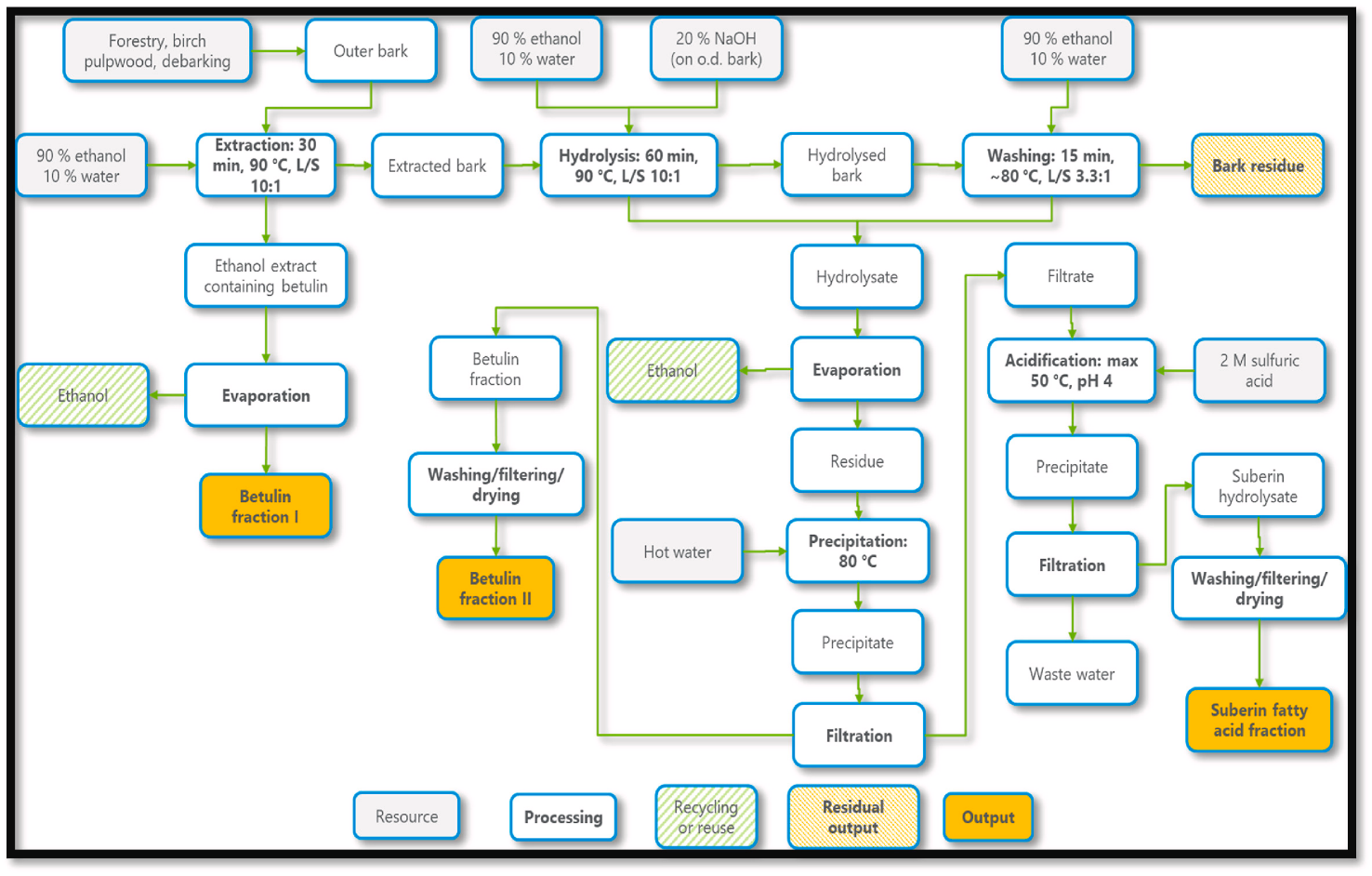
**

**Figure S2**: Production of suberin and betulin from outer bark of hard wood.

**Source:** (Yadav et al. 2024)

**Table S1:** Inventory of 1 m^2^ coating formation from Suberin, betulin and lignin, nanoparticle formation and application of coating on fabric.

| **Components** | **Inputs for Suberin, betulin and lignin coating for 1 m^2^ area** | **Unit** | **Reference or Remark** |
| --- | --- | --- | --- |
| Lignin, Betulin and Suberin each | 81.7 | gm |  |
| Acetone (conc 100 %) | 8170 | ml |  |
| Stirring for 1 hr | 1 | kWh | 1000 w stirrer used for 2 hr. |
| Stirring temperature (10 mint 85 ℃) | 0.65 | kcal | only betulin and suberin required |
| **Total** | **817** | **ml** |  |
| **NanoParticle formatoin (self assembly)** | | | |
| Water | 2860 | ml |  |
| Lignin solution | 817.14 | ml |  |
| Cellulose membrane for acetone removal | 2.87 | g/m2 | 130 mm Standard Flat Filter Paper Box of 100 flat standard qualitative paper filters of 130 mm diameter used with Buchners funnels weight 77 g/m² and thickness 0.16+0.18 mm. The retention pore is 8+11 µm micron: grade 2. |
| Acetone removal | 0.11 | kWh/kg | (Capello et al. 2005) |
| Final product (coating materials) | 2860 | ml | Coating solution |
| **Coating process on 1 m^2^ fabric** | | | |
| Ethanol | 572 | ml |  |
| Water | 1716 | ml |  |
| Cationic starch | 2860 | ml |  |
| Suberin, or Betulin or Lignin coating dispersion | 2860 | ml |  |

**Table S2:** Inventory of 1 kg untransformed dry kraft lignin powder (using natural gas) based on Ecoinvent 3.10.

| **Input** | **Unit** | **Quantity** | **ecoinvent process name** |
| --- | --- | --- | --- |
| Natural gas | MJ | 31.5 | Natural gas burned in industrial furnace >100 kW/RER |
| CO_2_ | kg | 0.30 | Carbon dioxide, liquid {RER}\| market for carbon dioxide, liquid \| Cut-off, U |
| H_2_SO_4_ | kg | 0.230 | Sulfuric acid {RER}\| market for sulfuric acid \| Cut-off, U |
| NaOH | kg | 0.107 | Sodium hydroxide, without water, in 50% solution state {RER}\| chlor-alkali electrolysis, membrane cell \| Cut-off, U |
| CaCO_3_ | kg | 0.230 | Limestone, crushed, for mill {RoW}\| limestone production, crushed, for mill \| Cut-off, U |
| Water | kg | 4.85 | Tap water {RER}\| market group for tap water \| Cut-off, U |
| Electricity | kWh | 0.010 | Electricity, medium voltage {FI}\| market for electricity, medium voltage \| Cut-off, U |
| Trucking | tkm | 0.934 | Transport, freight, lorry, 16-32 metric ton, diesel, EURO 6 {RER}\| market for transport, freight, lorry, 16-32 metric ton, diesel, EURO 6 \| Cut-off, U |

**Table S3**: Environmental prices for LCA: ReCiPe 2016 midpoints for the EU27, in € per unit.

| **Impact categories** | **Unit** | **Central Price*** |
| --- | --- | --- |
| Global warming potential (GWP) | €/kg CO_2_ eq. | 0.13 |
| Ionizing radiation (IR) | €/kBq Co-60 eq. | 0.00422 |
| Ozone formation (OF) | €/kg NOx eq. | 2.17 |
| Fine particulate matter formation (FPMF) | €/kg PM^2.5^ eq. | 99.2 |
| Terrestrial acidification (TA) | €/kg SO_2_ eq. | 5.27 |
| Freshwater eutrophication (FWE) | €/kg P eq. | 3.74 |
| Terrestrial ecotoxicity (TE) | €/kg 1.4-DCB | 0.00064 |
| Freshwater ecotoxicity (FE) | €/kg 1.4-DCB | 0.0209 |
| Marine ecotoxicity (ME) | €/kg 1.4-DCB | 0.0032 |
| Human carcinogenic toxicity (HCT) | €/kg 1.4-DCB | 3.99 |
| Human non carcinogenic toxicity (HNC) | €/kg 1.4-DCB | 0.071 |
| Land use (LU) | €/m^2^a crop eq. | 0.099 |
| Mineral resource scarcity (MRS) | €/kg Cu eq. | 0.014 |
| Fossil resource scarcity (FRS) | €/kg oil eq. | 0.028 |
| Water consumption (WC) | €/m^3^ | 0.407 |

**Source:** CE Delft (2024), *For use in LCAs it was recommend the central value because it contains the most likely outcome considering all uncertainties CE Delft (2024).

**Table S4:** Environmental impact different stages of suberin coating, betulin coating and lignin coating.

| Impact categories | Unit | Suberin Soln. | Betulin Soln. | Lignin Soln. | Nano+particle formation (suberin. betulin & lignin) | Application of coating (suberin. betulin & lignin) |
| --- | --- | --- | --- | --- | --- | --- |
| Global warming potential (GWP) | kg CO_2_ eq. | 1.373 | 0.843 | 0.466 | 0.613 | 0.931 |
| Ionizing radiation (IR) | kBq Co+60 eq. | 1.6282 | 0.9993 | 0.287 | 0.620 | 0.027 |
| Ozone formation (OF) | kg NOx eq. | 0.0018 | 0.0014 | 0.001 | 0.0012 | 0.0021 |
| Fine particulate matter formation (FPMF) | kg PM_2.5_ eq. | 0.0022 | 0.0009 | 0.000 | 0.0009 | 0.0009 |
| Terrestrial acidification (TA) | kg SO_2_ eq | 0.0062 | 0.0019 | 0.001 | 0.0018 | 0.0028 |
| Freshwater eutrophication (FWE) | kg P eq | 0.0005 | 0.0003 | 0.000 | 0.0002 | 0.0003 |
| Terrestrial ecotoxicity (TE) | kg 1.4-DCB | 7.5962 | 6.8691 | 6.862 | 1.8234 | 2.1350 |
| Freshwater ecotoxicity (FE) | kg 1.4-DCB | 0.0140 | 0.0081 | 0.003 | 0.0143 | 0.0067 |
| Marine ecotoxicity (ME) | kg 1.4-DCB | 0.0259 | 0.0171 | 0.010 | 0.0200 | 0.0087 |
| Human carcinogenic toxicity (HCT) | kg 1.4-DCB | 0.0312 | 0.0190 | 0.008 | 0.0268 | 0.0160 |
| Human non carcinogenic toxicity (HNC) | kg 1.4-DCB | 0.8061 | 0.4922 | 0.148 | 0.4965 | 0.2459 |
| Land use (LU) | m^2^a crop eq. | 0.2343 | 0.0753 | 0.021 | 0.0958 | 0.4741 |
| Mineral resource scarcity (MRS) | kg Cu eq. | 0.0015 | 0.0009 | 0.000 | 0.0013 | 0.0006 |
| Fossil resource scarcity (FRS) | kg oil eq. | 0.4378 | 0.2761 | 0.168 | 0.1626 | 0.5115 |
| Water consumption (WC) | m^3^ | 0.0532 | 0.0229 | 0.047 | 0.1916 | 0.0325 |

**Table S5**: Results of contribution analysis of different inputs used in the process to production of suberin coating per FU.

| **Impact category** | **Unit** | **Suberin** | **Electricity** | **Acetone** | **Water** | **Ethanol** | **Cationic starch** | **Cellulose filter** |
| --- | --- | --- | --- | --- | --- | --- | --- | --- |
| GWP | kg CO_2_ eq. | 1.1209 | 0.4215 | 0.1158 | 0.0330 | 0.6327 | 0.2974 | 0.2947 |
| IR | kBq Co+60 eq. | 1.3472 | 0.8568 | 0.0041 | 0.0172 | 0.0187 | 0.0078 | 0.0227 |
| OF | kg NOx eq. | 0.0013 | 0.0007 | 0.0002 | 0.0001 | 0.0014 | 0.0008 | 0.0007 |
| FPMF | kg PM^2.5^ eq. | 0.0019 | 0.0004 | 0.0001 | 0.0001 | 0.0004 | 0.0005 | 0.0006 |
| TA | kg SO_2_ eq. | 0.0057 | 0.0008 | 0.0002 | 0.0001 | 0.0010 | 0.0018 | 0.0011 |
| FWE | kg P eq. | 0.0004 | 0.0001 | 0.0000 | 0.0000 | 0.0002 | 0.0001 | 0.0001 |
| TE | kg 1.4-DCB | 1.2424 | 1.2294 | 5.9565 | 0.0821 | 0.7127 | 1.4181 | 0.9132 |
| FE | kg 1.4-DCB | 0.0120 | 0.0040 | 0.0008 | 0.0009 | 0.0021 | 0.0046 | 0.0107 |
| ME | kg 1.4-DCB | 0.0173 | 0.0064 | 0.0065 | 0.0013 | 0.0035 | 0.0052 | 0.0143 |
| HCT | kg 1.4-DCB | 0.0250 | 0.0111 | 0.0027 | 0.0053 | 0.0078 | 0.0081 | 0.0140 |
| HNC | kg 1.4-DCB | 0.6805 | 0.2885 | 0.0324 | 0.0382 | 0.1015 | 0.1438 | 0.2636 |
| LU | m^2^a crop eq. | 0.2142 | 0.0600 | 0.0007 | 0.0010 | 0.0046 | 0.4695 | 0.0542 |
| MRS | kg Cu eq. | 0.0012 | 0.0007 | 0.0000 | 0.0001 | 0.0001 | 0.0005 | 0.0007 |
| FRS | kg oil eq. | 0.3433 | 0.0971 | 0.0631 | 0.0092 | 0.4528 | 0.0586 | 0.0878 |
| WC | m^3^ | 0.0472 | 0.0171 | 0.0005 | 0.1777 | 0.0020 | 0.0287 | 0.0041 |

**Note:** Global warming potential (GWP), Ionizing radiation (IR), Ozone formation (OF), Fine particulate matter formation (FPMF), Terrestrial acidification (TA), Freshwater eutrophication (FWE), Terrestrial ecotoxicity (TE), Freshwater ecotoxicity (FE), Marine ecotoxicity (ME), Human carcinogenic toxicity (HCT), Human non-carcinogenic toxicity (HNC), Land use (LU), Mineral resource scarcity (MRS), Fossil resource scarcity (FRS), Water consumption (WC).

**Table S6**: Results of contribution analysis of different inputs used in the process to production of betulin coating per FU.

| **Impact category** | **Unit** | **Betulin** | **Electricity** | **Acetone** | **Water** | **Ethanol** | **Cationic starch** | **Cellulose filter** |
| --- | --- | --- | --- | --- | --- | --- | --- | --- |
| GWP | kg CO_2_ eq. | 0.591 | 0.422 | 0.116 | 0.033 | 0.633 | 0.297 | 0.295 |
| IR | kBq Co^+60^ eq. | 0.718 | 0.857 | 0.004 | 0.017 | 0.019 | 0.008 | 0.023 |
| OF | kg NOx eq. | 0.001 | 0.001 | 0.000 | 0.000 | 0.001 | 0.001 | 0.001 |
| FPMF | kg PM^2.5^ eq. | 0.001 | 0.000 | 0.000 | 0.000 | 0.000 | 0.001 | 0.001 |
| TA | kg SO_2_ eq. | 0.001 | 0.001 | 0.000 | 0.000 | 0.001 | 0.002 | 0.001 |
| FWE | kg P eq. | 0.000 | 0.000 | 0.000 | 0.000 | 0.000 | 0.000 | 0.000 |
| TE | kg 1.4-DCB | 0.515 | 1.229 | 5.956 | 0.082 | 0.713 | 1.418 | 0.913 |
| FE | kg 1.4-DCB | 0.006 | 0.004 | 0.001 | 0.001 | 0.002 | 0.005 | 0.011 |
| ME | kg 1.4-DCB | 0.009 | 0.006 | 0.007 | 0.001 | 0.003 | 0.005 | 0.014 |
| HCT | kg 1.4-DCB | 0.013 | 0.011 | 0.003 | 0.005 | 0.008 | 0.008 | 0.014 |
| HNC | kg 1.4-DCB | 0.367 | 0.289 | 0.032 | 0.038 | 0.102 | 0.144 | 0.264 |
| LU | m^2^a crop eq. | 0.055 | 0.060 | 0.001 | 0.001 | 0.005 | 0.469 | 0.054 |
| MRS | kg Cu eq. | 0.001 | 0.001 | 0.000 | 0.000 | 0.000 | 0.000 | 0.001 |
| FRS | kg oil eq. | 0.182 | 0.097 | 0.063 | 0.009 | 0.453 | 0.059 | 0.088 |
| WC | m^3^ | 0.017 | 0.017 | 0.000 | 0.178 | 0.002 | 0.029 | 0.004 |

**Note:** Global warming potential (GWP), Ionizing radiation (IR), Ozone formation (OF), Fine particulate matter formation (FPMF), Terrestrial acidification (TA), Freshwater eutrophication (FWE), Terrestrial ecotoxicity (TE), Freshwater ecotoxicity (FE), Marine ecotoxicity (ME), Human carcinogenic toxicity (HCT), Human non-carcinogenic toxicity (HNC), Land use (LU), Mineral resource scarcity (MRS), Fossil resource scarcity (FRS), Water consumption (WC).

**Table S7:** Results of contribution analysis of different inputs used in the process to production of lignin coating per FU.

| **Impact category** | **Unit** | **Lignin** | **Electricity** | **Acetone** | **Water** | **Ethanol** | **Cationic starch** | **Cellulose filter** |
| --- | --- | --- | --- | --- | --- | --- | --- | --- |
| GWP | kg CO_2_ eq. | 0.214 | 0.421 | 0.107 | 0.033 | 0.633 | 0.297 | 0.295 |
| IR | kBq Co+60 eq. | 0.007 | 0.857 | 0.004 | 0.017 | 0.019 | 0.008 | 0.023 |
| OF | kg NOx eq. | 0.000 | 0.001 | 0.000 | 0.000 | 0.001 | 0.001 | 0.001 |
| FPMF | kg PM^2.5^ eq. | 0.000 | 0.000 | 0.000 | 0.000 | 0.000 | 0.001 | 0.001 |
| TA | kg SO_2_ eq. | 0.000 | 0.001 | 0.000 | 0.000 | 0.001 | 0.002 | 0.001 |
| FWE | kg P eq. | 0.000 | 0.000 | 0.000 | 0.000 | 0.000 | 0.000 | 0.000 |
| TE | kg 1.4-DCB | 0.508 | 1.229 | 5.525 | 0.082 | 0.713 | 1.418 | 0.913 |
| FE | kg 1.4-DCB | 0.001 | 0.004 | 0.001 | 0.001 | 0.002 | 0.005 | 0.011 |
| ME | kg 1.4-DCB | 0.001 | 0.006 | 0.006 | 0.001 | 0.003 | 0.005 | 0.014 |
| HCT | kg 1.4-DCB | 0.002 | 0.011 | 0.002 | 0.005 | 0.008 | 0.008 | 0.014 |
| HNC | kg 1.4-DCB | 0.023 | 0.288 | 0.030 | 0.038 | 0.102 | 0.144 | 0.264 |
| LU | m^2^a crop eq. | 0.001 | 0.060 | 0.001 | 0.001 | 0.005 | 0.469 | 0.054 |
| MRS | kg Cu eq. | 0.000 | 0.001 | 0.000 | 0.000 | 0.000 | 0.000 | 0.001 |
| FRS | kg oil eq. | 0.074 | 0.097 | 0.059 | 0.009 | 0.453 | 0.059 | 0.088 |
| WC | m^3^ | 0.041 | 0.017 | 0.000 | 0.178 | 0.002 | 0.029 | 0.004 |

**Note:** Global warming potential (GWP), Ionizing radiation (IR), Ozone formation (OF), Fine particulate matter formation (FPMF), Terrestrial acidification (TA), Freshwater eutrophication (FWE), Terrestrial ecotoxicity (TE), Freshwater ecotoxicity (FE), Marine ecotoxicity (ME), Human carcinogenic toxicity (HCT), Human non-carcinogenic toxicity (HNC), Land use (LU), Mineral resource scarcity (MRS), Fossil resource scarcity (FRS), Water consumption (WC).

**Table S8:** Environmental impact changes in electricity from Finnish (FI), European (EU), Global (GLO) electricity mix).

| **Impact categories** | **Unit** | **Suberin GLO** | **Suberin**  **EU** | **Suberin FI** | **Betulin GLO** | **Betulin EU** | **Betulin FI** | **Lignin GLO** | **Lignin EU** | **Lignin FI** |
| --- | --- | --- | --- | --- | --- | --- | --- | --- | --- | --- |
| GWP | kg CO_2_ eq. | 4.740 | 3.475 | 2.916 | 4.210 | 2.945 | 2.386 | 4.151 | 2.886 | 2.336 |
| IR | kBq Co+60 eq. | 1.678 | 0.686 | 2.274 | 1.049 | 1.404 | 1.646 | 0.337 | 0.692 | 0.934 |
| OF | kg NOx eq. | 0.009 | 0.005 | 0.005 | 0.009 | 0.006 | 0.005 | 0.008 | 0.005 | 0.004 |
| FPMF | kg PM2.5 eq. | 0.008 | 0.003 | 0.004 | 0.007 | 0.004 | 0.003 | 0.006 | 0.003 | 0.002 |
| TA | kg SO2 eq | 0.017 | 0.008 | 0.011 | 0.013 | 0.009 | 0.006 | 0.012 | 0.008 | 0.005 |
| FWE | kg P eq | 0.002 | 0.001 | 0.001 | 0.002 | 0.002 | 0.001 | 0.002 | 0.001 | 0.001 |
| TE | kg 1.4-DCB | 12.174 | 10.309 | 11.555 | 11.447 | 10.825 | 10.828 | 11.009 | 10.386 | 10.820 |
| FE | kg 1.4-DCB | 0.065 | 0.042 | 0.035 | 0.059 | 0.048 | 0.029 | 0.053 | 0.043 | 0.024 |
| ME | kg 1.4-DCB | 0.096 | 0.064 | 0.055 | 0.087 | 0.072 | 0.046 | 0.079 | 0.065 | 0.039 |
| HCT | kg 1.4-DCB | 0.229 | 0.094 | 0.074 | 0.217 | 0.107 | 0.062 | 0.206 | 0.096 | 0.051 |
| HNC | kg 1.4-DCB | 3.060 | 1.675 | 1.548 | 2.746 | 2.042 | 1.235 | 2.400 | 1.695 | 0.891 |
| LU | m2a crop eq. | 0.773 | 0.563 | 0.804 | 0.614 | 0.618 | 0.645 | 0.560 | 0.564 | 0.591 |
| MRS | kg Cu eq. | 0.003 | 0.002 | 0.003 | 0.002 | 0.003 | 0.003 | 0.002 | 0.002 | 0.002 |
| FRS | kg oil eq. | 1.571 | 0.948 | 1.112 | 1.409 | 1.130 | 0.950 | 1.297 | 1.017 | 0.842 |
| WC | m3 | 0.273 | 0.232 | 0.277 | 0.243 | 0.249 | 0.247 | 0.268 | 0.274 | 0.271 |

**Note:** Global warming potential (GWP), Ionizing radiation (IR), Ozone formation (OF), Fine particulate matter formation (FPMF), Terrestrial acidification (TA), Freshwater eutrophication (FWE), Terrestrial ecotoxicity (TE), Freshwater ecotoxicity (FE), Marine ecotoxicity (ME), Human carcinogenic toxicity (HCT), Human non-carcinogenic toxicity (HNC), Land use (LU), Mineral resource scarcity (MRS), Fossil resource scarcity (FRS), Water consumption (WC).

**Table S9:** Environmental impact changes in electricity from Finnish electricity to Europe and global electricity.

**Note**: Global (GLO), Finnish (FI), European (EU), While **+** symbol denoting the increase in impact (%) while negative one for the decrease in the percentage.

| **Impact categories** | **Unit** | **Suberin** | | **Betulin** | | **Lignin** | |
| --- | --- | --- | --- | --- | --- | --- | --- |
|  |  | FI to GLO (%) | FI to EU (%) | FI to GLO (%) | FI to EU  (%) | FI to GLO (%) | FI to EU  (%) |
| GWP | kg CO_2_ eq. | +63 | +19 | +76 | +23 | +91 | +28 |
| IR | kBq Co^+60^ eq. | **-26** | **-70** | **-36** | **-15** | **-64** | **-26** |
| OF | kg NOx eq. | +84 | -7 | +90 | +20 | +109 | +24 |
| FPMF | kg PM^2.5^ eq. | +102 | 25 | +148 | +35 | +191 | +45 |
| TA | kg SO_2_ eq. | +57 | 30 | +95 | +38 | +114 | +46 |
| FWE | kg P eq. | +117 | +42 | +144 | +100 | +187 | +130 |
| TE | kg 1.4 DCB | +5 | 0.03 | +6 | 0.03 | +6 | 0.03 |
| FE | kg 1.4-DCB | +84 | +21 | +101 | +66 | +125 | +82 |
| ME | kg 1.4-DCB | +76 | +17 | +90 | +58 | +108 | +70 |
| HCT | kg 1.4-DCB | +210 | +28 | +252 | +73 | +307 | +90 |
| HNCT | kg 1.4-DCB | +98 | +8 | +122 | +65 | +170 | +91 |
| LU | m^2^a crop eq. | **-4** | **-30** | **-5** | **-4** | **-5** | **-5** |
| MRS | kg Cu eq. | **-13** | **-42** | **-16** | **-6** | **-20** | **-8** |
| FRS | kg oil eq. | +41 | -15 | +48 | +19 | +55 | +21 |
| WC | m^3^ | -1 | -16 | -2 | +1 | -1 | +1 |

**Note:** Global warming potential (GWP), Ionizing radiation (IR), Ozone formation (OF), Fine particulate matter formation (FPMF), Terrestrial acidification (TA), Freshwater eutrophication (FWE), Terrestrial ecotoxicity (TE), Freshwater ecotoxicity (FE), Marine ecotoxicity (ME), Human carcinogenic toxicity (HCT), Human non-carcinogenic toxicity (HNC), Land use (LU), Mineral resource scarcity (MRS), Fossil resource scarcity (FRS), Water consumption (WC).

**Table S10:** Environmental impact changes due to change of used of bioethanol instead of fossil ethanol.

| **Impact category** | **Unit** | **Suberin Ethanol** | **Suberin Bioethanol** | **Betulin ethanol** | **Betulin Bioethanol** | **Lignin Ethanol** | **Lignin Bioethanol** |
| --- | --- | --- | --- | --- | --- | --- | --- |
| GWP | kg CO_2_ eq. | 2.916 | 2.532 | 2.386 | 2.002 | 2.336 | 1.943 |
| IR | kBq Co+60 eq. | 2.274 | 2.264 | 1.646 | 1.635 | 0.934 | 0.923 |
| OF | kg NOx eq. | 0.005 | 0.005 | 0.005 | 0.004 | 0.004 | 0.003 |
| FPMF | kg PM^2.5^ eq. | 0.004 | 0.004 | 0.003 | 0.003 | 0.002 | 0.002 |
| TA | kg SO_2_ eq. | 0.011 | 0.012 | 0.006 | 0.007 | 0.005 | 0.006 |
| FWE | kg P eq. | 0.001 | 0.001 | 0.001 | 0.001 | 0.001 | 0.000 |
| TE | kg 1.4-DCB | 11.555 | 12.524 | 10.828 | 11.797 | 10.820 | 11.359 |
| FE | kg 1.4-DCB | 0.035 | 0.035 | 0.029 | 0.029 | 0.024 | 0.024 |
| ME | kg 1.4-DCB | 0.055 | 0.055 | 0.046 | 0.046 | 0.039 | 0.039 |
| HCT | kg 1.4-DCB | 0.074 | 0.068 | 0.062 | 0.056 | 0.051 | 0.045 |
| HNC | kg 1.4-DCB | 1.548 | 1.683 | 1.235 | 1.369 | 0.891 | 1.023 |
| LU | m^2^a crop eq. | 0.804 | 1.623 | 0.645 | 1.464 | 0.591 | 1.410 |
| MRS | kg Cu eq. | 0.003 | 0.003 | 0.003 | 0.003 | 0.002 | 0.002 |
| FRS | kg oil eq. | 1.112 | 0.717 | 0.950 | 0.555 | 0.842 | 0.443 |
| WC | m^3^ | 0.277 | 0.282 | 0.247 | 0.252 | 0.271 | 0.277 |

**Note:** Global warming potential (GWP), Ionizing radiation (IR), Ozone formation (OF), Fine particulate matter formation (FPMF), Terrestrial acidification (TA), Freshwater eutrophication (FWE), Terrestrial ecotoxicity (TE), Freshwater ecotoxicity (FE), Marine ecotoxicity (ME), Human carcinogenic toxicity (HCT), Human non-carcinogenic toxicity (HNC), Land use (LU), Mineral resource scarcity (MRS), Fossil resource scarcity (FRS), Water consumption (WC).

**Table S11**: External (environmental cost) of suberin, betulin and lignin coatings.

| **Impact categories** | **Unit** | **Suberin coating Production** | **Betulin coating Production** | **Lignin coating Production** | **Suberin coating per FU** | **Betulin coating per FU** | **Lignin coating per FU** |
| --- | --- | --- | --- | --- | --- | --- | --- |
| GWP | € | 0.2581 | 0.1892 | 0.1401 | 0.3791 | 0.3102 | 0.2612 |
| IR | € | 0.0095 | 0.0068 | 0.0038 | 0.0096 | 0.0069 | 0.0039 |
| OF | € | 0.0065 | 0.0065 | 0.0043 | 0.0109 | 0.0109 | 0.0087 |
| FPMF | € | 0.2976 | 0.1984 | 0.0992 | 0.3968 | 0.2976 | 0.1984 |
| TA | € | 0.0422 | 0.0211 | 0.0158 | 0.0580 | 0.0316 | 0.0264 |
| FWE | € | 0.0037 | 0.0000 | 0.0000 | 0.0037 | 0.0037 | 0.0037 |
| TE | € | 0.0060 | 0.0056 | 0.0056 | 0.0074 | 0.0069 | 0.0069 |
| FE | € | 0.0006 | 0.0005 | 0.0004 | 0.0007 | 0.0006 | 0.0005 |
| ME | € | 0.0001 | 0.0001 | 0.0001 | 0.0002 | 0.0001 | 0.0001 |
| HCT | € | 0.2314 | 0.1835 | 0.1397 | 0.2953 | 0.2474 | 0.2035 |
| HNCT | € | 0.0925 | 0.0702 | 0.0458 | 0.1099 | 0.0877 | 0.0633 |
| LU | € | 0.0327 | 0.0169 | 0.0116 | 0.0796 | 0.0639 | 0.0585 |
| MRS | € | 0.0000 | 0.0000 | 0.0000 | 0.0000 | 0.0000 | 0.0000 |
| FRS | € | 0.0168 | 0.0123 | 0.0093 | 0.0311 | 0.0266 | 0.0236 |
| WC | € | 0.0997 | 0.0871 | 0.0973 | 0.1127 | 0.1005 | 0.0973 |
| **External Total** | €/ kg | **1.10** | **0.80** | **0.57** | **1.50** | **1.20** | **0.96** |

**Note:** Global warming potential (GWP), Ionizing radiation (IR), Ozone formation (OF), Fine particulate matter formation (FPMF), Terrestrial acidification (TA), Freshwater eutrophication (FWE), Terrestrial ecotoxicity (TE), Freshwater ecotoxicity (FE), Marine ecotoxicity (ME), Human carcinogenic toxicity (HCT), Human non-carcinogenic toxicity (HNC), Land use (LU), Mineral resource scarcity (MRS), Fossil resource scarcity (FRS), Water consumption (WC).

**Table S12:** Production cost of suberin, betulin and lignin coating.

| **Components** | **unit** | **Cost** | **Amount** | **Amount as per production** | **Calculated cost** |
| --- | --- | --- | --- | --- | --- |
| Kraft lignin | €/kg | 0.380 | 81.7 | 0.0817 | 0.031 |
| Suberin | €/kg | 25 | 81.7 | 0.0817 | 2.043 |
| Betulin | €/kg | 13 | 81.7 | 0.0817 | 1.062 |
| Aceton | €/kg | 1.253 | 82 | 0.082 | 0.103 |
| Water | €/kg | 0.003 | 2860 | 2.86 | 0.007 |
| Electricity | €/kWh | 0.089 | 0.696 | 0.696 | 0.062 |
| **Application inputs** | | | | | |
| Ethanol | €/kg | 0.805 | 572 | 0.572 | 0.460 |
| Bioethanol | €/kg | 0.776 | 572 | 0.572 | 0.444 |
| **Total** | | | | | |
| Total Production cost of Lignin without application | | | | Euro per kg | 0.20 |
| Total Production cost of Betulin without application | | | | Euro per kg | 1.23 |
| Total Production cost of Suberin without application | | | | Euro per kg | 2.21 |
|  | | | | | |
| Production cost of Lignin with application (ethanol) | | | | Euro per kg | 0.663 |
| Production cost of Betulin with application (ethanol) | | | | Euro per kg | 1.694 |
| Production cost of Suberin with application (ethanol) | | | | Euro per kg | 2.675 |
|  | | | | | |
| Production cost of Lignin with application (bioethanol) | | | | Euro per kg | 0.647 |
| Production cost of Betulin with application (bioethanol) | | | | Euro per kg | 1.678 |
| Production cost of Suberin with application (bioethanol) | | | | Euro per kg | 2.658 |

**Table S13**: Results of uncertainty analysis.

|  | **Mean** | **Median** | **SD** | **CV** | **97.5 %** | **SEM** |
| --- | --- | --- | --- | --- | --- | --- |
| **Global warming** | | | | | | |
| Suberin coating | 2.92 | 2.90 | 0.15 | 5.26 | 3.62 | 0.00 |
| Betulin coating | 2.39 | 2.37 | 0.15 | 6.28 | 2.72 | 0.00 |
| Lignin coating | 2.00 | 1.98 | 0.15 | 7.52 | 2.34 | 0.00 |
| **Terrestrial ecotoxicity** | | | | | | |
| Suberin coating | 10.21 | 8.96 | 5.11 | 50.08 | **22.7** | 0.10 |
| Betulin coating | 10.3 | 9.0 | 5.1 | 49.20 | **23.1** | 0.10 |
| Lignin coating | 10.31 | 9.18 | 4.51 | 43.76 | **21.7** | 0.00 |
| **Land use** | | | | | | |
| Suberin coating | 0.59 | 0.58 | 0.12 | 20.73 | 0.863 | 0.001 |
| Betulin coating | 0.588 | 0.576 | 0.121 | 20.629 | 0.853 | 0.001 |
| Lignin coating | 0.59 | 0.58 | 0.12 | 20.47 | 0.858 | 0.001 |
| **Water consumption** | | | | | | |
| Suberin coating | 0.25 | 1.03 | 10.18 | 40.85 | 17.89 | 0.10 |
| Betulin coating | **0.33** | **1.09** | **10.08** | **30.10** | **17.73** | **0.10** |
| Lignin coating | 0.4 | 1.1 | 10.0 | 26.37 | 18.0 | 0.1 |
| **Ionizing radiation** | | | | | | |
| Suberin coating | 0.93 | 0.54 | 1.32 | 142.33 | 4.18 | 0.01 |
| Betulin coating | 0.94 | 0.53 | 1.44 | 153.53 | 4.03 | 0.01 |
| Lignin coating | 0.91 | 0.53 | 1.54 | 168.88 | 4.00 | 0.02 |
| **Human non-carcinogenic toxicity** | | | | | | |
| Suberin coating | -30.44 | -33.10 | 1250.39 | -4108.09 | 2442.72 | 39.54 |
| Betulin coating | 9.603 | 22.801 | 1249.036 | 13006.834 | 2468.630 | 12.490 |
| Lignin coating | -18.71 | -15.50 | 1247.74 | -6667.51 | 2471.44 | 12.48 |
